# Cultural and Gender Influences on Facial Attractiveness: A Comparative Study of Japanese and American Raters Using Geometric Morphometrics

**DOI:** 10.1002/pchj.70065

**Published:** 2025-11-15

**Authors:** Takanori Sano, Hideaki Kawabata

**Affiliations:** ^1^ Graduate School of Engineering The University of Tokyo Tokyo Japan; ^2^ Graduate School of Human Relations Keio University Tokyo Japan; ^3^ Faculty of Letters Keio University Tokyo Japan

**Keywords:** America, cultural differences, facial attractiveness, gender, Japan, Morphometrics

## Abstract

Facial attractiveness is a critical factor in forming interpersonal impressions. Evaluations of facial attractiveness were previously considered universal. However, it has recently been pointed out that individuals and cultures can diversify their evaluations. This study conducted Web experiments using the facial images of Japanese and American participants to examine the effects of raters' gender, age, and culture on facial attractiveness. Experiment 1 examined the impact of gender and age on Japanese raters. Experiment 2 explored the effects of culture on Japanese and American raters. Statistical and morphometric analyses were conducted on the obtained data. The results showed significant positive correlations between attractiveness ratings across gender, age, and culture. However, the results of the geometric morphometrics revealed that several differences in preferences regarding facial contours were observed among participants by gender. Additionally, Japanese raters were more likely than American raters to emphasize raised eyebrows for faces in attractive male images, and smaller mouths for faces in attractive female images. These results suggest that the facial features driving attractiveness evaluations differ depending on gender and culture, offering detailed insights into the culturally diverse standards of facial attractiveness. This study adds to the growing understanding of how cultural and individual factors shape aesthetic preferences, questioning the notion of universal beauty, and offering a clearer framework for future cross‐cultural research on facial attractiveness.

## 1 Introduction

Facial attractiveness is a crucial social interface in interpersonal relationships. Several studies have focused on this topic. Previous studies have emphasized that facial attractiveness is universal, as a high degree of agreement exists among most of the criteria for evaluating facial attractiveness, concerning which faces are rated as attractive (Cunningham et al. 1995; Langlois et al. 1991, 2000). However, individual differences exist according to gender (Cloutier et al. 2008; Little et al. 2001, 2002; Proverbio et al. 2010) and age (Ebner 2008; Foos and Clark 2011; He et al. 2021; Lin et al. 2020). In addition, evaluations may partly differ depending on race and culture (Coetzee et al. 2014; Pavlovič et al. 2023; Rhee and Lee 2010; Rhodes et al. 2005; Voegeli et al. 2021). Furthermore, these evaluation tendencies are diversified according to an individual's preferences and culture (Leder et al. 2016; Zhan et al. 2021).

Trends in attractiveness ratings may differ depending on the rater's gender and age. Regarding gender, differences have been reported between same‐ and opposite‐sex evaluations of attractiveness, as perceived from sexual dimorphism (Johnston 2006; Little et al. 2001, 2002). Some studies have reported that male faces with more masculine facial morphological features are rated as attractive (DeBruine et al. 2006; Little and Mannion 2006), whereas others have reported that male faces with more feminine facial morphology are rated as attractive (Little et al. 2001; Perrett et al. 1998). This discrepancy has been partly attributed to individual differences in women raters' preferences for masculine facial features (DeBruine et al. 2006; Johnston 2006; Marcinkowska et al. 2019). Similarly, attractiveness ratings may change depending on the raters' age. For example, facial attractiveness has been reported to decrease with increasing age of the facial image; however, the effect is less pronounced in judgments made by older perceivers than in those made by younger or middle‐aged perceivers (He et al. 2021).

These findings indicate that evaluations of attractiveness are not fixed but can vary depending on the perceiver's characteristics, such as age, gender, or other cultural and social factors. For example, Coetzee et al. (2014) found significant cross‐cultural agreement in facial attractiveness judgments between South African and Scottish participants, but this agreement was stronger for faces of familiar ethnicity, suggesting that perceptual experience influences cross‐cultural agreement. Rhodes et al. (2005) found that Caucasian participants rated own‐race composites as more attractive than other‐race composites, but only for male faces. However, mixed‐race (Caucasian/Japanese) composites were rated as even more attractive, suggesting that signs of health rather than prototypicality may better explain facial attractiveness. More recently, a large‐scale multi‐ethnic study conducted across five countries (China, France, India, Japan, and South Africa) showed that perceptions of female facial attractiveness are shaped not only by universal age‐related cues but also by the cultural background and gender of the rater as well as the ethnicity of the face being evaluated. This work highlighted both shared patterns and striking cultural differences, suggesting that attractiveness judgments are flexible rather than fixed (Voegeli et al. 2021). Similarly, a study examining evaluations of Vietnamese faces by Asian Vietnamese, Czech‐Vietnamese, and Czech‐European raters found broad agreement in overall attractiveness ratings but revealed that Czech‐Europeans placed strong emphasis on facial averageness as a cue to attractiveness. This suggests that while some cues are universally valued, the relative weight given to them can differ depending on cultural familiarity and exposure to particular facial types (Pavlovič et al. 2023).

Flexible methods are required to analyze these diverse evaluations of facial attractiveness in detail. Geometric morphometrics is one of the methods commonly used for examining the correspondence between morphological features and facial impressions (Abend et al. 2015; Boothroyd et al. 2013; Farrera et al. 2015; Rostovtseva et al. 2024; Windhager et al. 2011, 2018). Windhager et al. (2011) suggest that highly attractive and taller men have longer, narrower jaws, and wider or fuller lips. Furthermore, an asymmetric cap (i.e., an asymmetric inverted U‐shaped) relationship exists between body fat percentage and attractiveness (Windhager et al. 2018). The details of the dispersion trends in the ratings of facial attractiveness are visually confirmed using morphometric analysis. Although previous studies claim universality of facial attractiveness based on the high correlation between attractiveness ratings among raters, it may be possible to extract details of the differences in rating trends among raters by considering the dispersion trends of the rating details.

In this study, we investigated the effects of raters' age, gender, and cultural differences on facial morphological features related to attractiveness by conducting Web experiments with both Japanese and American participants. Japanese and American individuals have been reported to differ in their evaluations of the intensity of facial expressions (Matsumoto 1992; Matsumoto et al. 2002; Matsumoto and Ekman 1989). Therefore, we assumed that we could extract critical facial features to examine the cultural differences in facial attractiveness. In Experiment 1, we conducted a Web experiment with Japanese participants to investigate the effects of raters' age and gender on facial attractiveness ratings. In Experiment 2, we carried out a Web experiment with American participants. We compared these results with those of the Japanese participants in Experiment 1 to investigate the effects of culture on facial attractiveness ratings.

## 2 Experiment 1

We conducted Web experiments using QiQUMO, a platform operated by Cross Marketing Inc., Tokyo, Japan (https://qiqumo.jp/), which has a research panel of ~10 million registered participants. In Experiment 1, we investigated the effects of raters' age and gender on their evaluations of facial attractiveness with Japanese participants.

### 2.1 Methods

#### 2.1.1 Participants

We collected responses from 192 Japanese participants older than 20 years, all of whom, belonged to the Asian racial group. Participants with the same value for all responses were excluded from the data as not being diligent, and response data from 173 participants (85 men and 87 women, mean age 44.5 ± 16.4) were used in the analysis. The Ethics Committee of Japan approved this study in accordance with the Declaration of Helsinki (Approval Number: 230450000, Approval Date: January 31, 2024). Participants were requested to provide informed consent on the screen at the beginning of the experiment.

#### 2.1.2 Stimuli

As stimuli, we used 400 facial images from the Chicago Face Database (Ma et al. 2015), 50 each from Asian, Black, White, and Latino men and women, to include as diverse a set of faces as possible. All of these facial photographs were expressionless and facing forward, and the photographic conditions were controlled. These facial images were selected in descending order of age distribution from the average by race and sex. The mean apparent age of the selected images was 27.95 years (SD = 3.82). A full list of apparent age values for all stimulus images is provided in the data repository indicated under the Data Availability statement. The original images in the Chicago Face Database had a resolution of 2444 × 1718 pixels, but we adjusted them to 1222 × 859 pixels because of the size limit for uploading to the QiQUMO platform.

As the analysis was based on the number of facial images, we determined that a sample size of 400 would be sufficient, because a power analysis had indicated that ~314 samples were necessary to achieve adequate statistical power (0.8) for detecting medium effect sizes (partial $\eta^{2}$ = 0.06) in a four‐factor ANOVA, including the age and gender of the raters, and sex and race of the facial images.

#### 2.1.3 Procedure

The experiment was divided into four experimental patterns, and each participant indicated the attractiveness of 100 facial images on a 7‐point scale (1 = *not at all attractive* to 7 = *very attractive*). This allocation was designed to obtain as many facial image evaluation scores as possible while considering the response burden per person. Each set of 100 images included a balanced number of faces across all race and sex categories (i.e., ~12–13 images from each of the 8 categories: Asian male, Asian female, Black male, Black female, White male, White female, Latino male, and Latino female). To minimize potential biases across the four stimulus sets, we confirmed that the mean attractiveness and age ratings (based on the Chicago Face Database normative scores) did not differ significantly across sets (attractiveness: ANOVA, *F* (3, 396) = 1.114, *p* = 0.343; age: ANOVA, *F* (3, 396) = 0.513, *p* = 0.674). Additionally, the age and sex distributions of each set were compared, and no significant differences were observed (age: ANOVA, *F* (3, 236) = 0.625, *p* = 0.599; sex: χ^2^ tests, *χ* ^2^ (3) = 0.6565, *p* = 0.883). Thus, the sets were comparable in stimulus properties and participant demographics. The presentation order of images within each set was counterbalanced for every participant. Additionally, participants were randomly assigned to one of the four sets, such that each set was evaluated by a comparable number of raters with diverse demographic characteristics. The proportions of facial image categories (sex and race) assigned to these four sets were similar. The experiment was conducted using each participant's personal computer or smartphone. There was no time limit for answering. After excluding participants who provided inappropriate or irregular responses, each facial image was evaluated by ~43 raters on average.

### 2.2 Analysis

#### 2.2.1 Basic Statistical Analysis

To confirm the effects of the gender and age of the raters, and the sex and race of the facial images, we calculated the frequency distribution of the average attractiveness scores for each facial image, aggregated by the gender and age of the rater (over and under 40), to confirm the overall rating trend. In addition, we calculated the correlations between the average attractiveness score of each facial image by the gender and age of the raters. As the distribution of mean attractiveness scores approximated normality, correlation analyses were conducted using Pearson's correlation.

#### 2.2.2 ANOVA

We conducted an ANOVA to examine the effects of facial attractiveness ratings. The analysis was based on the average attractiveness score for each facial image, as well as the rater's gender and age, and the sex and race of the facial image. A mixed‐design ANOVA was used to analyze the variance effects and to examine each factor. The ANOVA was not conducted to discuss preferences for the facial characteristics of specific racial groups, but to examine the preference tendencies for the selected facial stimuli and provide complementary insights to the morphometric results. These statistical analyses were carried out in R (version 4.4.1).

#### 2.2.3 Morphometrics Analysis

We conducted a morphometric analysis to investigate detailed trends in the relationship between attractiveness ratings and facial morphological features. To prepare for the analysis, 68 facial landmarks were automatically assigned to each facial image using Python with a LIB library. Next, we used all 65 landmark points, excluding three that were densely located around the mouth, for smooth visualization. Procrustes analysis (Kendall 1984) was used to minimize the distance from the reference, and the center of gravity was aligned as the reference position of the landmark points. Warps were then calculated from the landmark points using thin‐plate splines, and fragments between landmarks were concatenated and smoothed. Warps correspond to changes in facial morphology. Multivariate regression analysis was conducted for male and female images, using warps as the dependent variable, and attractiveness scores as the independent variables. In other words, we constructed a regression model showing the changes in shape information linked to the attractiveness score. In the morphometric regression analyses, attractiveness ratings were used as predictors of shape variation to visualize the morphological trends associated with higher or lower ratings. This procedure follows standard applications of geometric morphometrics (e.g., Rohlf 2021a) and is intended as a descriptive mapping of covariation between shape and ratings. In the analysis, the scores of the participants' answers were standardized by considering individual differences in the scale of responses. The average of the standardized scores was calculated as the attractiveness score for each image and further standardized to match the scale between images. Standardization was calculated, such that, the mean was 0 and variance was 1. This operation was performed on both the male and female images. We used the Generalized Goodall *F*‐test and permutation test (1000 permutations) to compute each regression model's statistical significance (*p*‐values) of the explained variance. Next, landmark locations and facial images were visualized. We used tpsRelw (version 1.75) to standardize the landmark points (Rohlf 2021b), tpsRegr (version 1.50) for the regression analysis and visualization of the landmark points (Rohlf 2021a), and tpsSuper (version 2.06) for imaging (Rohlf 2021c).

The primary aim of the morphometric analysis was not to investigate the racial categories of the face stimuli but rather to examine how the cultural background of raters influenced perceptions of attractiveness. To ensure a sufficient sample size for the morphometric models and to capture overall tendencies, facial images were pooled across racial categories, excluding race as a factor. However, to provide complementary insights, ANOVAs were conducted separately to analyze the effects of race on attractiveness ratings.

### 2.3 Results

#### 2.3.1 Results of Basic Statistical Analysis

**FIGURE 1:**
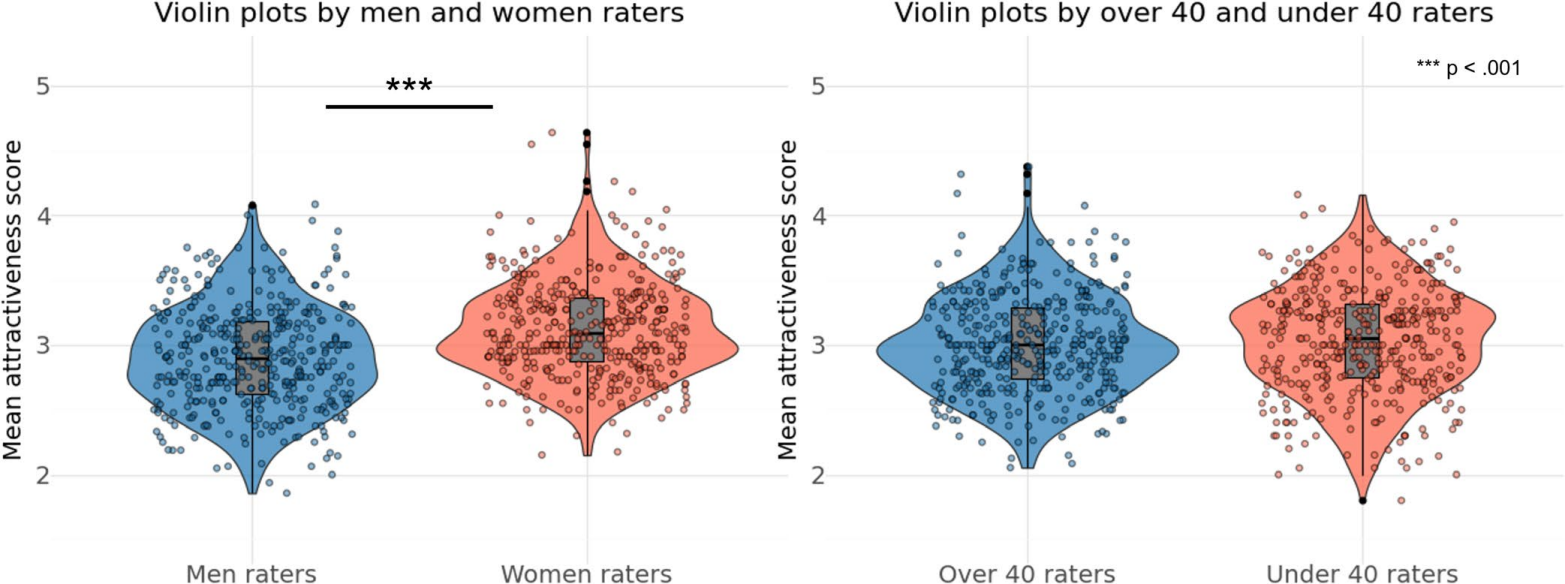
Violin plots by gender and age of the raters. This violin plot illustrates the distribution of the mean scores for each facial image, aggregated by men and women raters and by raters over and under 40 years. The central boxplot within each violin displays the median (thick central line), interquartile range (bounds of the box), and minimum and maximum values (whiskers). The width of the violin represents data density, with wider sections indicating a higher concentration of scores within that range. The overlaid scatter points depict the mean rating scores for each facial image, averaged within each rater group.

**FIGURE 2:**
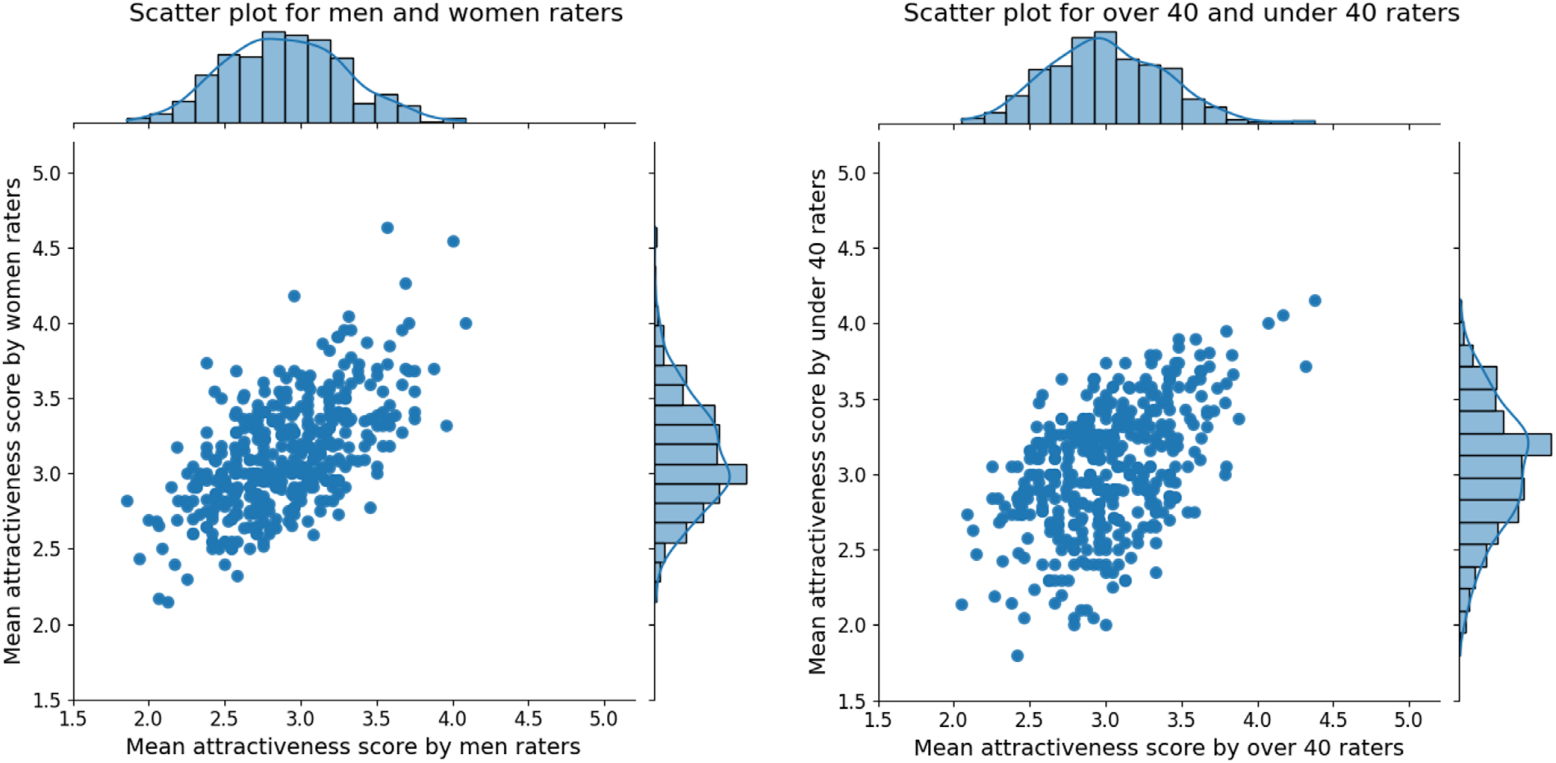
Scatter plots by gender and age of the raters. These scatter plots show the relationship between mean attractiveness scores given by different rater groups. Each point represents the mean score for a facial image, with marginal histograms indicating the score distributions for each group.

We calculated the frequency distribution of the average attractiveness scores for each facial image, aggregated by raters' gender and age (over and under 40 years), to confirm the overall rating trend. The results are shown in Figure 1. The t‐test results showed that women rated attractiveness scores higher than men (*t*(399) = 12.925, *p* < 0.001, *d* = 0.659). No significant differences by age were observed between the groups (*t*(399) = 1.498, *p* = 0.110, *d* = 0.065). In addition, correlations were calculated for the average attractiveness score of each facial image by gender and age of the rater. A positive and significant correlation (*r* = 0.605, *p* < 0.001) was obtained for the gender of the rater (between men and women), and a positive and significant correlation (*r* = 0.497, *p* < 0.001) was obtained for the age of the rater (over and under 40 years). The results are shown in Figure 2.

#### 2.3.2 Results of ANOVA

We conducted an ANOVA to examine the effects of facial attractiveness ratings. It was based on the average attractiveness score for each facial image, gender and age of the raters, and sex and race of the facial image. The mixed‐design ANOVA analyzed the effects of each impact. Full statistical results, including all main effects and interactions, are summarized in Table 1, with effect sizes reported. In the text, we highlight the significant effects and interactions.

**TABLE 1:** Summary of ANOVA results and effect sizes for Experiment 1.

Effect	df	*F* value	*p*‐value	Partial $\eta^{2}$
Race of the facial image	3, 392	5.730	< 0.001	0.042
Sex of the facial image	1, 392	1.512	0.220	0.004
Raters' age	1, 392	0.483	0.487	0.001
Raters' gender	1, 392	3.925	< 0.05	0.010
Race × Sex	3, 392	1.512	0.348	0.008
Race × Age	3, 392	1.430	0.234	0.011
Sex × Age	1, 392	0.097	0.755	< 0.001
Race × Sex × Age	3, 392	0.584	0.625	0.005
Age × Gender	1, 392	319.867	< 0.001	0.449
Race × Gender	3, 392	1.863	0.135	0.014
Sex × Gender	1, 392	2.730	0.099	0.007
Race × Sex × Gender	3, 392	1.543	0.203	0.012
Race × Age × Gender	3, 392	1.603	0.188	0.012
Sex × Age × Gender	1, 392	0.007	0.934	< 0.001
Race × Sex × Age × Gender	3, 392	0.353	0.787	0.003

**FIGURE 3:**
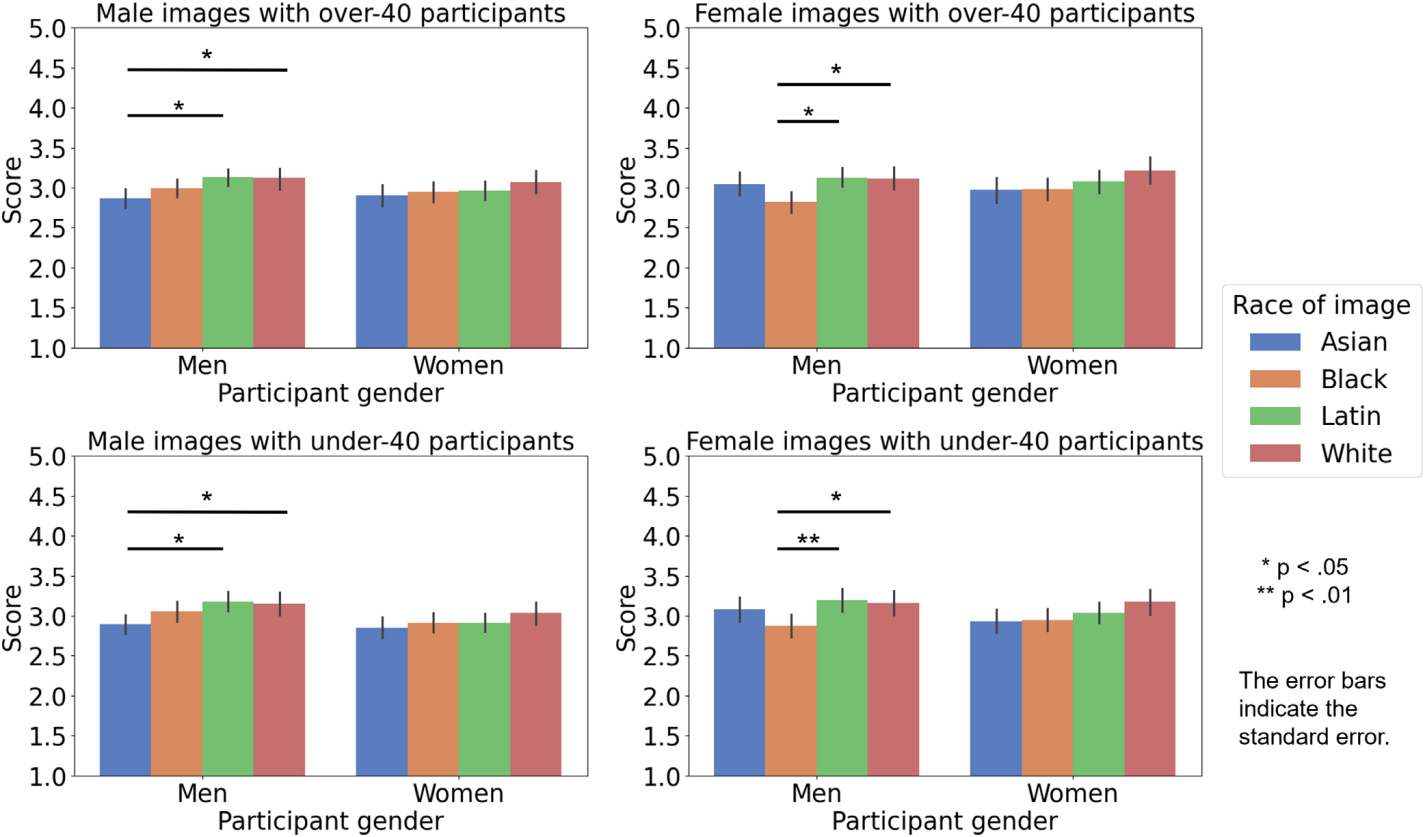
Average attractiveness scores by age and gender of the raters, and race and sex of the facial images.

The ANOVA results indicated significant main effects of race of images (*F*(3, 392) = 5.730, *p* < 0.001, partial $\eta^{2}$ = 0.042) and raters' gender (*F*(1, 392) = 3.925, *p* < 0.05, partial $\eta^{2}$ = 0.010). Significant interactions were observed between the rater's age and gender (*F*(1, 392) = 319.867, *p* < 0.001, partial $\eta^{2}$ = 0.449). Post hoc pairwise comparisons of the race of images based on gender, age of raters, and sex of image were conducted. The results are shown in Figure 3.

The analysis revealed that the gender and age of the rater, and the sex and race of the facial image affect attractiveness, with complex interactions between them. This dataset indicated that White images tended to be rated as relatively attractive.

#### 2.3.3 Results of Morphometrics Analysis

Multivariate regression analysis revealed that specific components of facial shape variation were significantly associated with participants' attractiveness ratings for male images (men over‐40 raters, women over‐40 raters, men under‐40 raters, and women under‐40 raters models: explained 2.44%, 1.61%, 2.57%, and 1.64% of variance, *p*s < 0.001 of Generalized Goodall *F*‐test, *p*s < 0.05 of 1000 permutation tests), and female images (men over‐40 raters, women over‐40 raters, men under‐40 raters, and women under‐40 raters models: explained 7.26%, 4.07%, 7.27%, and 4.06% of variance, *p* < 0.001, *p*s < 0.001, *p* < 0.001 of Generalized Goodall *F*‐test, *p*s < 0.01 of 1000 permutation tests).

**FIGURE 4:**
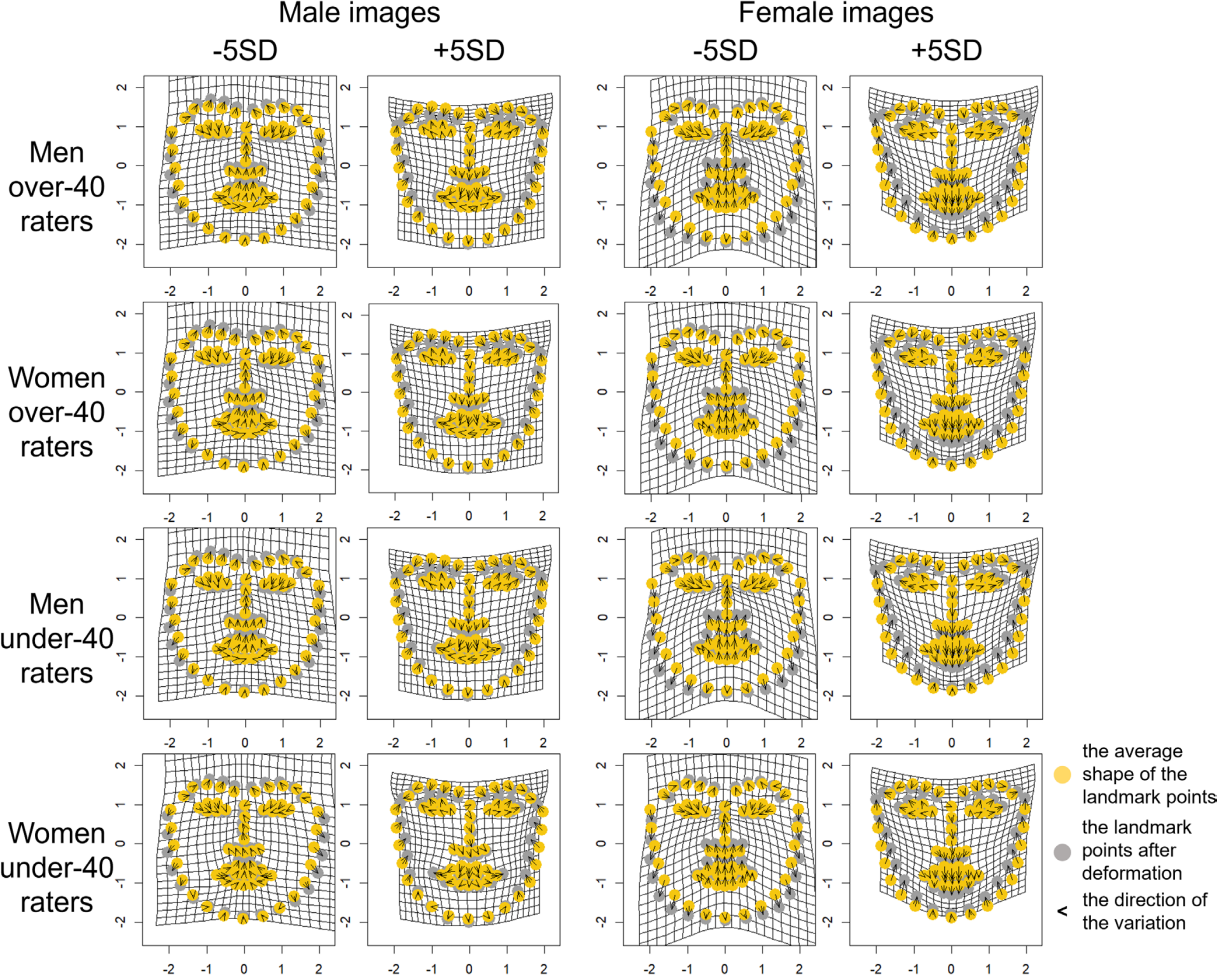
Results of geometric morphometrics of facial images by Japanese raters.

**FIGURE 5:**
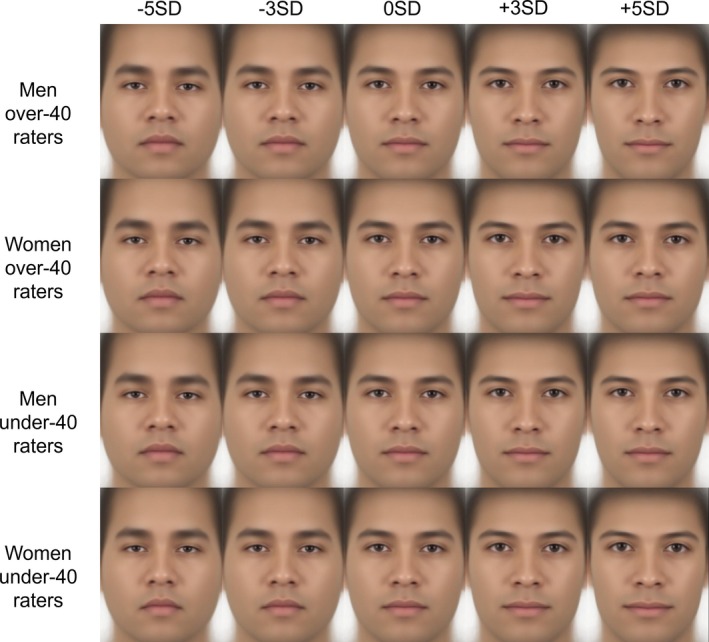
Results of geometric morphometrics of male facial images by Japanese raters.

**FIGURE 6:**
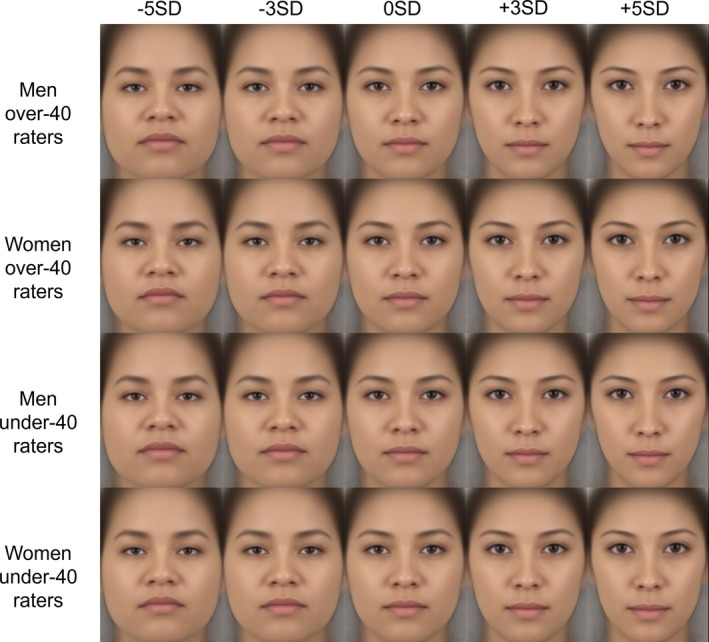
Results of geometric morphometrics of female facial images by Japanese raters.

The obtained ratings were then used to generate facial images that highlighted facial features related to attractiveness, using geometric morphometrics to investigate the features corresponding to attractiveness. Visualization showed that in male images, men raters tended to prefer longer noses, sharper jawlines, and raised eyebrows when facial features corresponding to attractiveness were emphasized. Women raters showed similar tendencies as men raters but preferred relatively more angular facial structure. Both men and women raters tended to prefer raised eyebrows and angular outlines in female images. A few differences were observed depending on gender and age. The details are presented in Figures 4, 5, and 6. Low (−5 SD, −3 SD) indicates the result when the attractiveness ratings are manipulated toward −SD, and High (+3 SD, +5 SD) indicates the result when the attractiveness ratings are manipulated toward +SD.

These results suggest that many facial features are commonly associated with attractiveness, but minor differences in male images may depend mainly on the rater's gender.

### 2.4 Discussion

The ANOVA results suggest that facial features related to facial attractiveness may differ depending on the gender and age of the raters. This finding is consistent with previous research, as gender (Little et al. 2001, 2002) and age (He et al. 2021; Lin et al. 2020) influence attractiveness ratings. The relatively higher attractiveness ratings for images of White faces may have been influenced by sexual dimorphism and the skin tone cues associated with it (Lewis 2011; Stephen et al. 2018). The results of geometric morphometrics show that attractive faces in both male and female images tend to have longer noses, sharper jawlines, and more raised eyebrows. However, women raters find relatively angular facial structures more attractive, particularly for male images. Perceived facial adiposity is a cue to health and an important factor in facial attractiveness (De Jager et al. 2018). Previous studies using geometric morphometrics have shown that a moderate BMI (Body Mass Index) is an important indicator of attractiveness (Windhager et al. 2018). Health cues are considered important indicators of mate choice (Rhodes 2006; Thornhill and Gangestad 1999; Little et al. 2011), so these results may have been obtained when evaluating the opposite sex. However, the geometric morphometric results indicated that age‐related differences were relatively small. Therefore, in the next experiment, in addition to the raters' gender, which shows a difference in the effect, we focused on the raters' culture.

## 3 Experiment 2

To collect data from American raters, we conducted Web experiments using the Global QiQUMO platform, operated by Cross Marketing Inc., Tokyo, Japan (https://qiqumo.jp/global_qiqumo/), which has a research panel of more than 20 million registered American participants. In Experiment 2, we compared the results of the Japanese participants from Experiment 1 with those of American participants. This study aimed to clarify the influence of raters' culture and gender on evaluating facial attractiveness.

### 3.1 Methods

#### 3.1.1 Participants

We collected responses from American participants older than 20 years, who belonged to either the White or Black racial group, and 282 participants' responses were included. Participants who had the same value for all responses were excluded from the data as not being diligent, and data from 270 participants' responses (144 men and 126 women, mean age 42.5 ± 13.1) were used in the analysis. The Ethics Committee of Japan approved this study in accordance with the Declaration of Helsinki (Approval Number: 230450000, Approval Date: January 31, 2024). Participants were requested to provide informed consent on the screen at the beginning of the experiment.

#### 3.1.2 Stimuli

The Stimuli Were 160 Facial Images From the Chicago Face Database, 20 Each of Asian, Black, White, and Latino Male and Female. These Facial Images Were Selected in Descending Order of Age Distribution From the Average by Race and Sex. These Images Were Selected From Those Used in Experiment 1. The Mean Apparent Age of the Selected Images Was 27.35 Years (SD = 3.23). A Full List of Apparent Age Values for all Stimulus Images Is Provided in Data Availability. The Original Images in the Chicago Face Database Had a Resolution of 2444 × 1718 Pixels but Were Adjusted to 1222 × 859 Pixels Because of the Size Limit for Uploading to the Global QiQUMO Platform.

Based on the large interaction effect between rater age and gender observed in Experiment 1, we hypothesized that rater factors (culture and gender) would similarly exhibit a large effect size in Experiment 2. Accordingly, as the analysis was based on the number of facial images, we determined that a sample size of 160 would be sufficient, because a power analysis indicated that approximately 131 samples were necessary to achieve adequate statistical power (0.8) for detecting large effect sizes (partial $\eta^{2}$ = 0.14) in a four‐factor ANOVA, including rater culture, rater gender, image sex, and image race.

#### 3.1.3 Procedure

The experiment was divided into four experimental patterns, and each participant indicated the attractiveness of 40 facial images on a 7‐point scale (1 = *not at all attractive* to 7 = *very attractive*). Similar to Experiment 1, this assignment was designed to obtain as many facial image evaluation scores as possible while considering the response burden per person. Each set of 40 images included an equal number of faces from each race and sex category (i.e., Asian, Black, White, and Latino; male and female), with 5 images per category, ensuring that participants evaluated a diverse and balanced set of faces. To minimize potential biases across the four stimulus sets, we confirmed that the mean attractiveness and age ratings (based on the Chicago Face Database normative scores) did not differ significantly across sets (attractiveness: ANOVA, *F* (3, 156) = 0.399, *p* = 0.754; age: ANOVA, *F* (3, 156) = 0.293, *p* = 0.830). Additionally, the age and sex distributions of each set were compared, and no significant differences were observed (age: ANOVA, *F* (3, 283) = 0.054, *p* = 0.983; sex: χ^2^ tests, *χ* ^2^ (3) = 0.366, *p* = 0.947). Thus, the sets were comparable in stimulus properties and participant demographics. Image order was counterbalanced within the sets for each participant, and participants were randomly assigned to sets to achieve counterbalancing across demographic groups. The proportions of facial image categories (sex and race) assigned to these four sets were similar. The experiment was conducted using each participant's personal computer or smartphone. There was no time limit for answering. After excluding participants who provided inappropriate or irregular responses, each facial image was evaluated by ~67 raters on average.

### 3.2 Analysis

The procedure was the same as that used in Experiment 1. We conducted basic statistical analysis, ANOVA, and morphometric analysis to confirm the effects of the gender and culture of the raters and the sex and race of the facial images. To examine the differences between the tendencies of Japanese and American raters, we analyzed the results from Experiments 1 and 2 for the 160 images that were used in both experiments.

As in Experiment 1, an image's race‐related characteristic was not included as a factor in the morphometric analysis to maintain sufficient sample size per condition. While ANOVA detected differences across races, this analysis focused on how such differences might be reflected in shared facial shape features.

### 3.3 Results

#### 3.3.1 Results of Basic Statistical Analysis

**FIGURE 7:**
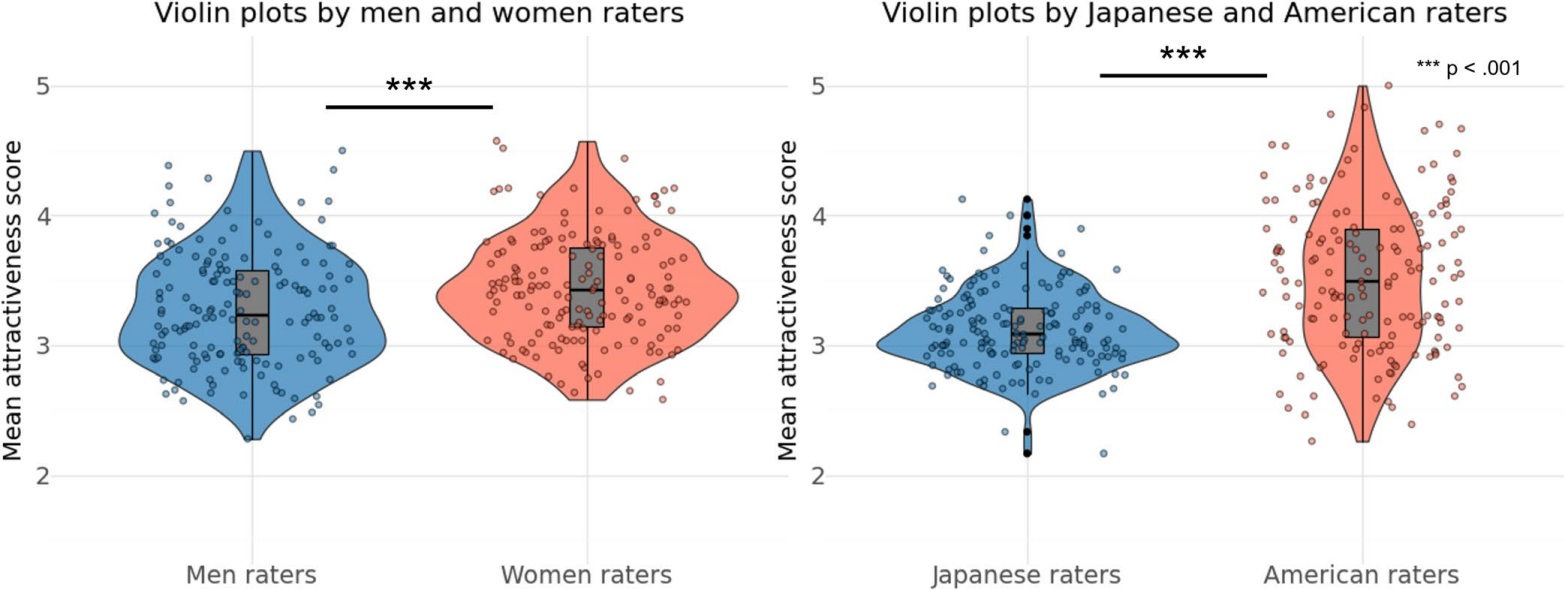
Violin plots by gender and culture of the raters. This violin plot illustrates the distribution of the mean scores for each facial image, aggregated by men and women raters and by Japanese and American raters. The central boxplot within each violin displays the median (thick central line), interquartile range (bounds of the box), and minimum and maximum values (whiskers). The width of the violin represents data density, with wider sections indicating a higher concentration of scores within that range. The overlaid scatter points depict the mean rating scores for each facial image, averaged within each rater group.

**FIGURE 8:**
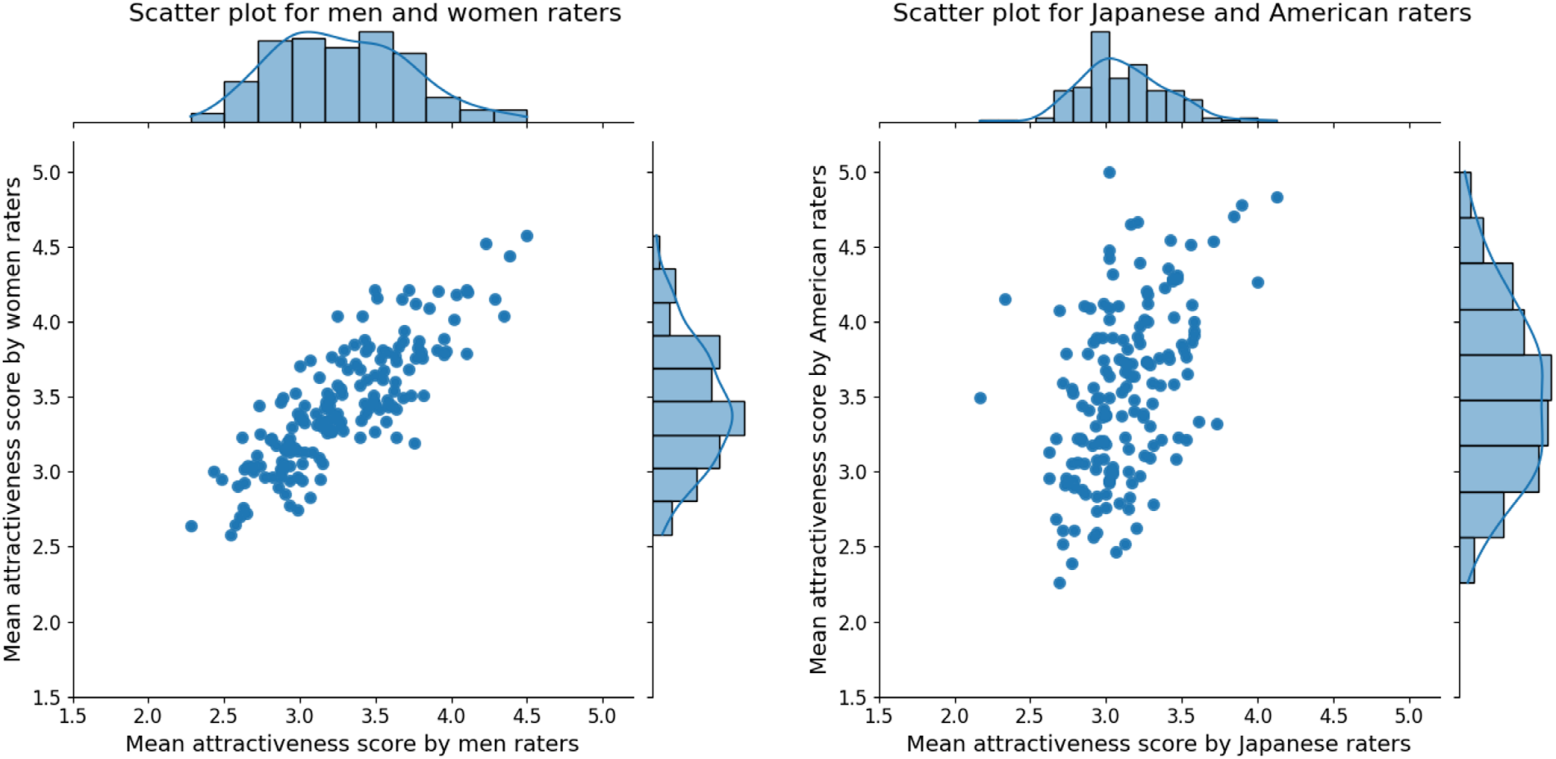
Scatter plots by gender and culture of the raters. These scatter plots show the relationship between mean attractiveness scores given by different rater groups. Each point represents the mean score for a facial image, with marginal histograms indicating the score distributions for each group.

We calculated the frequency distribution of the average attractiveness score for each facial image aggregated by rater gender and culture to confirm the overall rating trend. The results are shown in Figure 7. The results of the *t*‐test showed that women raters rated attractiveness scores higher than men raters (*t*(159) = 8.575, *p* < 0.001, *d* = 0.673). American raters tended to give higher ratings than Japanese raters (*t*(159) = 9.890, *p* < 0.001, *d* = 0.787). Japanese raters gave relatively moderate scores (SD = 0.30), while American raters gave relatively broad scores (SD = 0.57). In addition, correlations were calculated between the mean attractiveness scores of each facial image by gender and age of the rater. We found a positive and significant correlation (*r* = 0.831, *p* < 0.001) for the rater's gender (between men and women) and a positive and significant correlation (*r* = 0.465, *p* < 0.001) for the rater's culture (between Japanese and American). The results are shown in Figure 8.

#### 3.3.2 Results of ANOVA

We conducted an ANOVA to examine the effects of facial attractiveness ratings. The analysis was based on the average attractiveness score for each facial image, the rater's gender and culture, and the sex and race of the facial image. The mixed‐design ANOVA analyzed the effects of each impact. In this subsection, we report the significant main effects and interactions from the ANOVA results.

The ANOVA revealed significant main effects on attractiveness ratings of images based on gender, culture of raters, and sex, race of image. Several interaction effects were also significant, including two‐, three‐, and four‐way interactions among these factors. Full statistical results and effect sizes are presented in Table 2. Notably, a significant four‐way interaction (race × gender × culture × gender) suggests that attractiveness judgments were influenced by complex combinations of participant and image characteristics.

**TABLE 2:** Summary of ANOVA results and effect sizes for Experiment 2.

Effect	df	*F* value	*p*‐value	Partial $\eta^{2}$
Race of the facial image	3, 152	9.73	< 0.001	0.161
Sex of the facial image	1, 152	135.73	< 0.001	0.472
Raters' culture	1, 152	229.21	< 0.001	0.601
Raters' gender	1, 152	108.96	< 0.001	0.418
Race × Sex	3, 152	2.85	< 0.05	0.053
Race × Culture	3, 152	1.99	0.117	0.038
Sex × Culture	1, 152	195.19	< 0.001	0.562
Race × Sex × Culture	3, 152	4.32	< 0.01	0.079
Culture × Gender	3, 152	0.082	0.774	0.001
Race × Gender	3, 152	10.27	< 0.001	0.169
Sex × Gender	1, 152	1.14	0.288	0.007
Race × Sex × Gender	3, 152	5.16	< 0.01	0.093
Race × Culture × Gender	3, 152	6.39	< 0.001	0.112
Sex × Culture × Gender	1, 152	14.78	< 0.001	0.089
Race × Sex × Culture × Gender	3, 152	3.3	< 0.05	0.061

**FIGURE 9:**
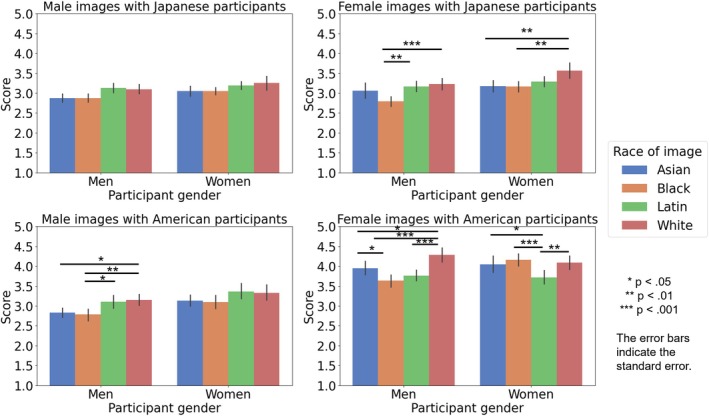
Average attractiveness scores by culture and gender of the raters, and race and sex of the facial images.

As in Experiment 1, post hoc pairwise comparisons were conducted focusing on the simple main effect of image based on race to examine which racial groups were perceived as more attractive, within each combination of images based on gender, culture of raters, and sex of image. For example, these comparisons revealed, that Japanese women rated White female faces significantly lower than Asian and Black female faces, whereas American women tended to rate Latino female faces significantly higher than other racial groups. The detailed results are shown in Figure 9.

The analysis revealed that the gender and culture of the rater, and sex and race of the facial image affect attractiveness, with complex interactions in each. Overall, White facial images were rated as more attractive.

#### 3.3.3 Results of Morphometric Analysis

Multivariate regression analysis revealed that specific components of facial shape variation were significantly associated with participants' attractiveness ratings for male images (rater models—Japanese men, Japanese women, American men, and American women—explained 2.28%, 1.10%, 1.74%, and 1.21% of variance, *p* < 0.001, *p* = 0.850, *p* < 0.001, and *p* = 0.620 of Generalized Goodall *F*‐test, *p* = 0.098, *p* = 0.479, *p* = 0.178, and *p* = 0.390 of 1000 permutation tests, respectively), and female images (rater models—Japanese men, Japanese women, American men, and American women—explained 5.32%, 2.86%, 2.05%, and 1.49% of variance, *p* < 0.001, *p* < 0.001, *p* < 0.001, and *p* = 0.085 of Generalized Goodall *F*‐test, *p* < 0.05, *p* < 0.05, *p* = 0.134, and *p* = 0.277 of 1000 permutation tests, respectively). In the Generalized Goodall *F*‐test, significant effects were observed for the Japanese men and American men rater models in male images, and for the Japanese men, Japanese women, and American men rater models in female images. The Japanese women rater model for male images did not reach statistical significance; however, its visualization revealed patterns broadly consistent with those observed in Experiment 1. We therefore interpreted these results cautiously in light of the earlier findings. By contrast, the American women rater models were not significant for either male or female images. Accordingly, while we inspected and interpreted the visualizations of these models to explore potential shape‐related tendencies, we also considered the possibility that factors other than morphological features contributed to attractiveness judgments, as well as the reasons why models based on men raters reached significance whereas those based on women raters did not.

**FIGURE 10:**
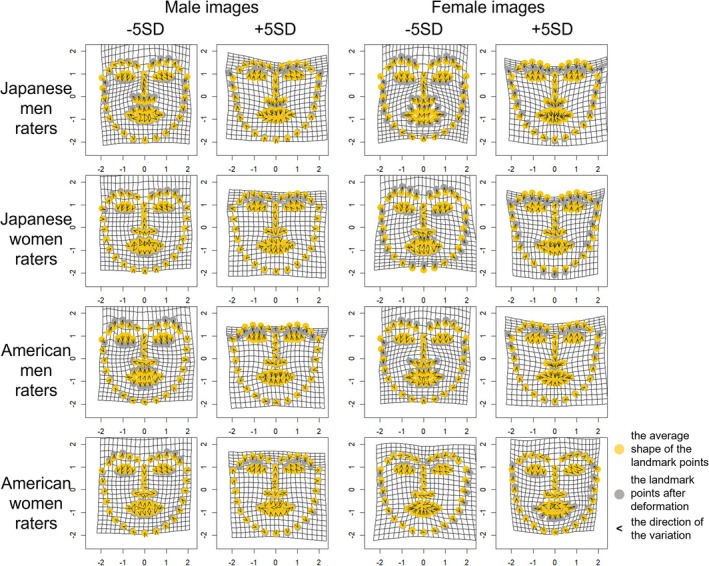
Results of geometric morphometrics of facial images by Japanese and American raters.

**FIGURE 11:**
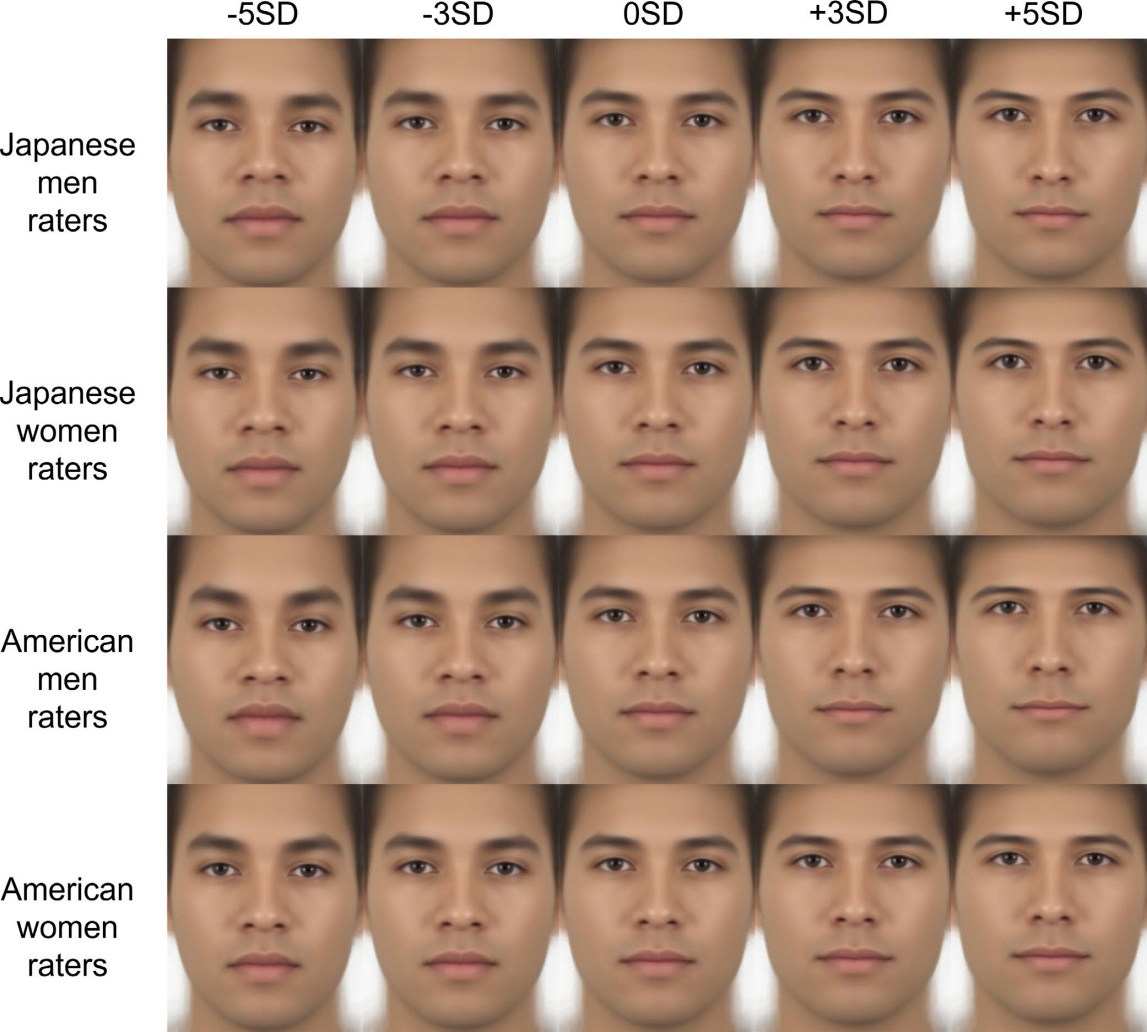
Results of geometric morphometrics of male facial images by Japanese and American raters.

**FIGURE 12:**
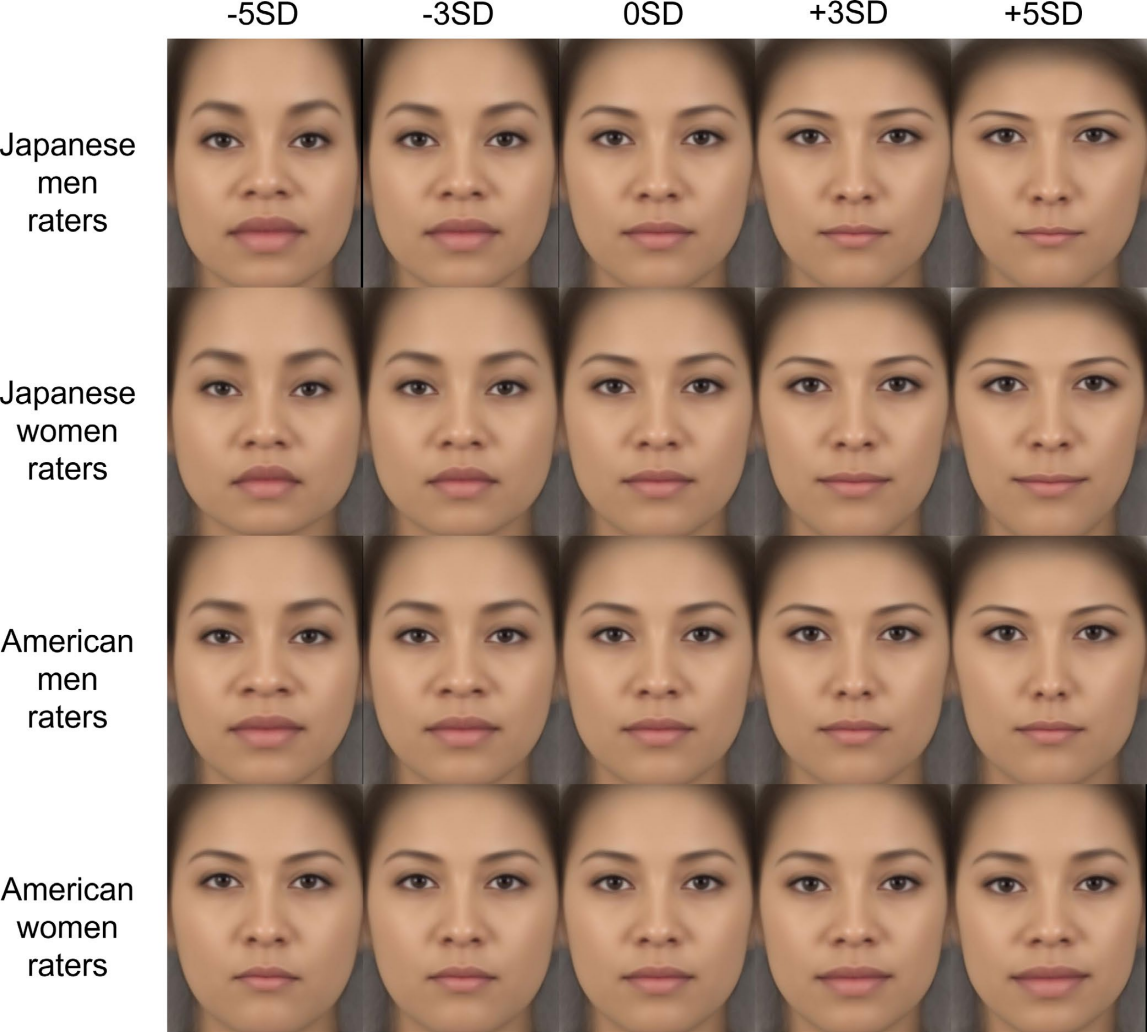
Results of geometric morphometrics of female facial images by Japanese and American raters.

The obtained ratings were used to generate facial images that highlighted facial features related to attractiveness using geometric morphometrics to investigate the features corresponding to attractiveness. Visualization showed that for male images, Japanese men raters tended to prefer longer noses, sharper jawlines, and raised eyebrows when facial features corresponding to attractiveness are emphasized. Japanese women raters tended to prefer larger eyes and an angle of eyebrows lower than that of Japanese men raters. These results aligned with those in Experiment 1. American men raters tended to prefer faces with low‐angled eyebrows and small mouths. American women raters tended to prefer faces with low‐angled eyebrows and larger mouths than American men raters. For female images, Japanese men and women raters tended to prefer faces with sharp jawlines, and small mouths and noses. These results tended to align with those of Experiment 1. American men raters tended to prefer faces with small noses and mouths. American women raters tended to prefer faces with wider eyebrows and relatively large mouths. The results are shown in Figures 10, 11, and 12. Low (−5 SD, −3 SD) indicates the result when the attractiveness ratings are manipulated toward −SD, and High (+3 SD, +5 SD) indicates the result when the attractiveness ratings are manipulated toward +SD.

These results indicate that the facial features associated with attractiveness differ depending on the rater's gender and culture.

### 3.4 Discussion

Japanese raters assigned moderate ratings with limited variance, whereas American raters assigned extreme ratings with greater variance. Previous research on rating the emotional intensity of facial images indicates that Americans provide more intense ratings than Japanese raters (Matsumoto 1992; Matsumoto et al. 2002; Matsumoto and Ekman 1989). Consequently, for facial impressions such as attractiveness, American raters may have more definitively assessed whether a face was attractive or unattractive. The ANOVA results suggest that facial features related to facial attractiveness may differ depending on the culture and gender of the rater. Hence, both the culture and gender of the rater are crucial factors that influence attractiveness ratings, consistent with previous studies (Coetzee et al. 2014; Little et al. 2001, 2002; Pavlovič et al. 2023; Rhee and Lee 2010; Rhodes et al. 2005; Voegeli et al. 2021). Images of White faces may have higher attractiveness ratings due to sexual dimorphism and the skin tone cues associated with it (Lewis 2011; Stephen et al. 2018). Further morphometric analysis revealed differences in facial features related to attractiveness.

In the case of male images, American raters tended to emphasize features, such as a small mouth and lowered eyebrows more than the Japanese raters. These features may be related to the link between angry expressions and dominance impressions (Montepare and Dobish 2003) and may have been influenced by the cultural aspects of communicating with others. In addition, among Japanese raters, men raters tended to prefer longer noses, sharper jawlines, and raised eyebrows. Women raters tended to prefer larger eyes and an angle of eyebrows lower than that of Japanese men raters. These features may be related to the sexual dimorphism traits of masculinity and femininity. Regarding the effect of masculinity on male faces, some studies have reported that faces with more masculine facial morphological features are rated as attractive (DeBruine et al. 2006; Little and Mannion 2006), whereas others have reported that male faces with more feminine facial morphology are rated as attractive (Little et al. 2001; Perrett et al. 1998), which has been partly attributed to individual differences in the preferences of women raters (DeBruine et al. 2006; Johnston 2006; Marcinkowska et al. 2019). The results of this study may also reveal complex relationships between the genders.

In the case of female images, large eyes, and sharp angular contours were important for attractiveness among Japanese raters, which aligns with previous studies (Sano and Kawabata 2023). In addition, we found a difference in raters' gender among the American raters. Women raters tended to prefer faces with larger noses, mouths, and wider eyebrows than men raters. This preference may be related to estrogen‐enhanced sexual dimorphism (Rhodes 2006; Thornhill and Gangestad 1999; Little et al. 2011).

The visualization results suggested that American women tended to prefer relatively broader facial shapes in both male and female faces. However, these models did not reach statistical significance in either the Generalized Goodall *F*‐test or the permutation test, and thus the findings should be regarded as exploratory and interpreted with caution. One possible explanation is that attractiveness judgments among American women rely not only on morphological cues but also on non‐shape‐based information such as skin texture (Jones et al. 2004) and coloration (de Lurdes Carrito et al. 2016), which were not captured by our morphometric analysis. Moreover, while the models based on men reached statistical significance, those based on women did not. This discrepancy may reflect individual differences in women's preferences for male facial masculinity, as reported in previous studies (DeBruine et al. 2006; Johnston 2006; Marcinkowska et al. 2019). Additionally, the lack of significance observed in specifically the American women models may be partly attributable to cultural and geographic diversity. Indeed, analyses based on data from all 50 U.S. states demonstrated that women's preferences for male facial masculinity can be systematically explained by state‐level health indices (DeBruine et al. 2010). These findings suggest that, compared with Japan, greater regional diversity in the United States may amplify heterogeneity in attractiveness judgments. The non‐significant results for American women in our study could thus reflect the combined effects of individual heterogeneity and regional variation. Nevertheless, from the perspective of robustness, the limited number of stimuli and participants in this study must be acknowledged as limitations. Therefore, further research is needed to examine these issues in greater detail. Consequently, our main interpretations were based on the models that reached statistical significance in Experiments 1 and 2, while the non‐significant models were treated as exploratory indications of potential contributions from non‐morphological factors, and interpreted with caution.

## 4 General Discussion and Conclusions

Across two web‐based experiments, we found that while attractiveness judgments were positively correlated across age, gender, and culture, the specific morphological features associated with attractiveness varied according to rater characteristics. These results suggest that attractiveness evaluation involves not only universal tendencies but also those shaped by cultural and social influences.

In the correlation analysis of Experiments 1 and 2, a positive correlation, independent of gender, age, and culture, was consistent with the results of previous studies (Cunningham et al. 1995; Langlois et al. 1991, 2000). Therefore, facial attractiveness has universal elements regarding the comparative agreement of relative ratings. However, although there was a significant correlation in each case, there were differences in ratings by culture in Experiment 2. When the frequency distribution of the ratings was calculated, Japanese raters gave relatively moderate ratings, whereas American raters gave relatively broad ratings. In previous studies on rating the emotional intensity of facial images, American raters gave more intense ratings than Japanese raters (Matsumoto 1992; Matsumoto et al. 2002; Matsumoto and Ekman 1989). Therefore, in the case of facial impressions such as attractiveness, American raters may have evaluated more clearly whether the face was attractive or unattractive in the same way.

We examined the relationship between facial features and attractiveness by visualizing facial features using geometric morphometry. The overall trend was consistent with previous studies that used geometric morphometrics to relate facial attractiveness to features, such as long noses and sharp jawlines (Sano and Kawabata 2023; Sano et al. 2024; Windhager et al. 2011, 2018). However, different trends were observed in terms of gender and culture. Women tended to show a somewhat greater emphasis on relatively angular facial structure for attractive male faces than men. In terms of culture, American raters tended to emphasize features, such as a small mouth and lowered eyebrows more than Japanese raters for male images. These results suggest that mate selection and cultural background may be related to attractiveness.

Differences have been reported in the gender of participants, especially in same‐sex and opposite‐sex ratings (Johnston 2006; Little et al. 2001, 2002). Regarding the effect of masculinity on male faces, some studies have reported that faces with more masculine facial morphological features are rated as attractive (DeBruine et al. 2006; Little and Mannion 2006), whereas others have reported that male faces with more feminine facial morphological features are rated as attractive (Little et al. 2001; Perrett et al. 1998). This discrepancy has been partly attributed to individual differences in women raters' preferences for masculine facial features (DeBruine et al. 2006; Johnston 2006; Marcinkowska et al. 2019). Masculine facial features are often interpreted as signals of genetic fitness and dominance, reflecting hormonal influences on facial development (Johnston 2006), whereas feminine features may indicate prosociality and higher parental investment (Kruger 2006; Moore et al. 2009; Perrett et al. 1998). In the present study, women were more likely than men to emphasize relatively angular facial structures when rating male faces as attractive. This tendency might reflect evolved mate preferences, possibly shaped by trade‐offs between perceived dominance and nurturance. Additionally, gender‐based preferences could be influenced by culturally socialized gender roles, which may shape ideals of desirable traits in romantic partners. These findings suggest that both biological and cultural factors may contribute to gender differences in facial attractiveness judgments. Furthermore, specific facial features, such as facial adiposity, may have influenced attractiveness ratings, particularly for female faces. Although we did not include objective health measures, such as BMI, or subjective health assessments, previous studies suggest that facial morphology can influence perceived health, and consequently, attractiveness (Little et al. 2011; Thornhill and Gangestad 1999; Rhodes 2006). Therefore, although this relationship cannot be directly verified in this study's data, it is plausible that some of the observed preferences were based on implicit health‐related impressions of facial structure. Future research may benefit from considering this potential influence more explicitly.

The observed cultural differences in preferred facial features may reflect broader cultural differences in perceptual and aesthetic orientations. In this study, Japanese raters—particularly women—showed a preference for relatively angular facial structures and spatial configurations, suggesting a more configural processing style, consistent with previous findings (Miyamoto et al. 2011). This tendency supports the view that the Japanese are more likely than Americans to perceive facial similarity based on a holistic configuration rather than individual feature matches. These preferences may also reflect culturally specific ideals of femininity. For instance, the Japanese preference for neotenous features, such as smaller noses and mouths, may align with prior evidence that Japanese men exhibit strong preferences for facial femininity compared with other cultures (Marcinkowska et al. 2014). Such preferences may stem either from cultural aesthetics that associate youthfulness with beauty, or evolutionary tendencies to value cues linked to reproductive health and lower dominance in long‐term mate selection (Rhodes 2006; Thornhill and Gangestad 1999; Little et al. 2011). Moreover, these culturally shaped preferences relate to differences in how facial information is processed in social contexts. Americans have been shown to rely more on the mouth than eyes when interpreting facial expressions, even under conditions such as mask‐wearing (Saito et al. 2023), which may relate to their heightened sensitivity to changes in mouth size observed in this study. Taken together, these findings underscore how culturally grounded perceptual strategies, and aesthetic ideals jointly shape the evaluation of facial attractiveness. In addition, the lack of significance in the American women models may reflect the influence of cultural and geographic diversity, as women's preferences for male facial masculinity have been shown to vary systematically across U.S. states according to state‐level health indices (DeBruine et al. 2010). However, clarifying the specific mechanisms underlying such regional variation will require further investigation in future research.

This study found little difference in the results based on the raters' age. Previous studies have reported that the older the age of the facial image to be evaluated, the less attractive it is perceived to be; however, this effect is more significant for young and middle‐aged people than for older people (He et al. 2021). In other words, there is likely to be an interaction between the evaluator's age and the face being evaluated. In this study, the age range of the facial images prepared as experimental stimuli was limited; therefore, large differences may not have been observed. In future studies, to investigate the differences by age, a more comprehensive age range of facial images as stimuli is expected.

The results of this study may depend on the specific set of stimuli used; however, this possibility is a common concern in research employing natural face photographs. Nevertheless, the Chicago Face Database (Ma et al. 2015) provides facial images that were photographed under standardized conditions and are relatively homogeneous, thereby minimizing such concerns. Moreover, compared with previous studies that relied on datasets of only a few dozen facial images (e.g., Windhager et al. 2011, 2018), our study is better able to capture variability in facial appearance through a larger image set. In addition, our findings showed many points of consistency with previous research (Sano and Kawabata 2023; Sano et al. 2024; Windhager et al. 2011, 2018), further reinforcing the validity of our results.

This study contributes to expanding the knowledge that the perception of facial attractiveness is not necessarily universal, but depends on the perceiver's characteristics, and is diverse. However, despite its contributions, this study has several limitations. First, as it focused on morphological features and used geometric morphometrics, factors such as skin texture and color were not examined. In future research, more advanced approaches, such as investigations using deep learning and machine learning, are expected (Sano 2022, 2023, 2025; Sano and Kawabata 2023, 2024; Sano et al. 2024). Second, its data‐driven modeling approach may depend on the features of the facial images used in the analysis. Further validation and analysis using extensive datasets are required. Third, the statistical significance of some models was not confirmed in Experiment 2. Therefore, additional facial images will be necessary for subsequent studies to emphasize the robustness of the model results. Fourth, we did not collect information about the racial or ethnic backgrounds of the American participants owing to practical constraints. Given the diversity of the U.S. population, this limits our ability to examine potential own‐race or ingroup biases. Previous research suggests that cultural and ethnic backgrounds can influence attractiveness judgments through differences in familiarity and perceptual strategies (Coetzee et al. 2014), which future studies should account for. Fifth, because of differences in Web survey platforms and fees, we could not set the number of images to be answered under the same conditions for the experiments targeting Japanese and American participants. Finally, the number of images evaluated by each participant in this study was limited because of participants' burden. In addition, to collect ratings for as many facial images as possible, the participant‐rated image sets were divided into several patterns. Rating experiments should use a single pattern with sufficient images in the future to consider the details of dispersion trends among raters (e.g., Leder et al. 2016).

## Ethics Statement

The Ethics Committee of Japan approved this study in accordance with the Declaration of Helsinki (Graduate School of Human Relations, Keio University, Approval Number: 230450000, Approval Date: January 31, 2024).

## Consent

Informed consent was obtained on the screen when the responses began in Experiments 1 and 2.

## Conflicts of Interest

The authors declare no conflicts of interest.

## Data Availability

The data that support the findings of this study are openly available in OSF at https://osf.io/a5wrv/.
